# Code status orders in hospitalized patients with COVID-19

**DOI:** 10.1016/j.resplu.2023.100452

**Published:** 2023-08-23

**Authors:** Amber R. Comer, Lyle Fettig, Stephanie Bartlett, Shilpee Sinha, Lynn D'Cruz, Aubrey Odgers, Carly Waite, James E. Slaven, Ryan White, Amanda Schmidt, Laura Petras, Alexia M. Torke

**Affiliations:** a Indiana University School of Health and Human Science, United States; b Indiana University School of Medicine, United States; c American Medical Association, United States

**Keywords:** COVID-19, Code Status

## Abstract

**Background:**

The COVID-19 pandemic created complex challenges regarding the timing and appropriateness of do-not-attempt cardiopulmonary resuscitation (DNACPR) and/or Do Not Intubate (DNI) code status orders. This paper sought to determine differences in utilization of DNACPR and/or DNI orders during different time periods of the COVID-19 pandemic, including prevalence, predictors, timing, and outcomes associated with having a documented DNACPR and/or DNI order in hospitalized patients with COVID-19.

**Methods:**

A cohort study of hospitalized patients with COVID-19 at two hospitals located in the Midwest. DNACPR code status orders including, DNI orders, demographics, labs, COVID-19 treatments, clinical interventions during hospitalization, and outcome measures including mortality, discharge disposition, and hospice utilization were collected. Patients were divided into two time periods (early and late) by timing of hospitalization during the first wave of the pandemic (March–October 2020).

**Results:**

Among 1375 hospitalized patients with COVID-19, 19% (*n* = 258) of all patients had a documented DNACPR and/or DNI order. In multivariable analysis, age (older) *p* =< 0.01, OR 1.12 and hospitalization early in the pandemic *p* = 0.01, OR 2.08, were associated with having a DNACPR order. Median day from DNACPR order to death varied between cohorts *p* => 0.01 (early cohort 5 days versus late cohort 2 days). In-hospital mortality did not differ between cohorts among patients with DNACPR orders, *p* = 0.80.

**Conclusions:**

There was a higher prevalence of DNACPR and/or DNI orders and these orders were written earlier in the hospital course for patients hospitalized early in the pandemic versus later despite similarities in clinical characteristics and medical interventions. Changes in clinical care between cohorts may be due to fear of resource shortages and changes in knowledge about COVID-19.

## Background

The COVID-19 pandemic created complex challenges regarding the timing and appropriateness of Do-Not-Attempt Cardiopulmonary Resuscitation (DNACPR) code status orders, including when and if it was appropriate to change a patient’s code status.^1^, ^2^, ^3^, ^4^, ^5^, ^6^, ^7^, ^8^, ^9^, ^10^, ^11^, ^12^, ^13^, ^14^, ^15^, ^16^, ^17^, ^18^, ^19^, ^20^ Code status orders indicate whether resuscitation would be attempted in the event of cardiac arrest as well as whether a patient should be intubated if they are experiencing respiratory distress. Ideally, code status decisions are made through a process of shared decision making between the physician and the patient and/or their surrogate medical decision maker.^15^, ^16^, ^17^, ^18^, ^19^, ^20^, ^21^ Patient or surrogate preferences should be informed by an understanding of prognosis of underlying health conditions and acute illness as well as outcomes from cardiopulmonary resuscitation, mechanical ventilation, and other critical care interventions.^21^, ^22^, ^23^ However, physicians frequently miss opportunities for shared decision-making through failure to engage in goals of care conversations and as such, code status does not always align with patients’ primary stated values.^21^, ^22^, ^23^, ^24^ In the intensive care unit, patients frequently lack capacity to make decisions, and surrogates are often called upon to make code status decisions.^24^

The management of COVID-19 critical illness as well as the understanding of prognosis and outcomes of resuscitation in the setting of COVID-19 evolved quickly during the first year of the pandemic, adding to the complexity of code status decisions. Initial reports may have overestimated mortality in mechanically ventilated patients.^24^, ^25^ The assumption of high mortality in the critical care setting led some to initially question the role of CPR in patients with COVID-19.^24^, ^25^, ^26^ Later data confirmed the limited effectiveness of CPR in critically ill patients with COVID-19, but clarified that outcomes were not universally poor, with younger age being associated with better outcomes.^24^, ^25^, ^26^ In addition, the risk of COVID-19 transmission during aerosolizing procedures brought forth a unique ethical challenge due to the need to balance the risk of spreading COVID-19 to health care workers during resuscitation efforts with the need to care for individual patients.

Due to the complex issues COVID-19 presented, a better understanding of the changes in utilization of code status orders during different time periods of the Covid-19 pandemic is needed. In order to address this gap in the literature we conducted a study to determine if there were differences in code status orders among patients hospitalized with COVID-19 during two different time periods of the pandemic in two hospitals. The time periods included the first three months of the pandemic when hospitals were faced with the new public health emergency and the second three months of the pandemic when cases had initially ebbed. Additionally, we examined the prevalence, predictors, timing, and outcomes associated with having a documented DNACPR and/or DNI order in these two cohorts of hospitalized patients with COVID-19.

## Methods

### Design and setting

A retrospective chart review of hospitalized patients with COVID-19 was performed to determine characteristics, medical treatments received, and outcomes associated with having a documented DNACPR and/or DNI order. Patients hospitalized with a diagnosis of COVID-19 at two hospitals located in the Midwest, both academic medical centers, were identified from the electronic medical record by an affiliated biomedical informatics institute (Regenstrief Institute, Indianapolis, Indiana). Both hospitals are urban, level one trauma centers, with one hospital having 625 beds and the other 333 beds. One hospital is the safety net hospital which mainly serves the uninsured. The hospitals admitted similar number of COVID-19 patients with Hospital 1 admitting *n* = 701 and Hospital 2 admitting *n* = 674 during the study period. Neither hospital included in this study evoked crisis standards during the pandemic. The University Institutional Review Board (IRB) approved this study.

### Participants

This study included patients with a confirmed COVID-19 infection hospitalized between March 2020 and October 2020. COVID-19 infection was considered confirmed if the patient had a positive nasopharyngeal sample by polymerase chain reaction (PCR) within two weeks prior to or during the index hospitalization. We also looked at primary discharge diagnosis to identify patients being treated for COVID-19.

### Data collection

Data extracted from the Electronic Medical Record (EMR) indicated code status orders including full code, Do Not Attempt Cardiopulmonary Resuscitation (DNACPR), Do Not Intubate (DNI) or a combination of DNACPR/DNI. A standardized chart review tool was created which gathered patient demographic information, life sustaining medical treatments received including ventilator, ICU admission, dialysis, and extracorporeal membrane oxygenation (ECMO). Additional medical care such as prone position and palliative care consultation were collected. Outcome measures including mortality, discharge disposition, and hospice utilization were collected. Chart reviewers were trained using a standardized chart review manual which included step-by-step instructions about where to locate the study variables in the patient medical record. All chart reviewers completed the same 20 charts and achieved at least 90% agreement on all variables collected.

### Statistical analysis

Patients were divided into two time periods (early and late) by timing of hospitalization during the first wave of the COVID-19 pandemic. The early cohort included patients whose hospital admission began during the months of March – June 2020. The late cohort was defined as patients whose hospital admission began during the months of July – October 2020.

Bivariate analyses were performed to determine associations between patient and clinical characteristics and the presence of a documented DNACPR and/or DNI order in the patient medical record. Student’s t-tests were used on normally distributed variables, Wilcoxon rank-sum on skewed continuous variables, and Chi-Square tests on categorical variables, using Fisher’s Exact tests to verify results when cell counts were small. A multivariable analysis was then performed using logistic regression models, with variables selected a-priori, including age, gender, race, and ethnicity, as well as using variables which reached an a priori cut-point of *p* < 0.20. Interaction effects were also tested between race and early/late cohort on having a DNACPR order or not, to ensure there were no racial changes between the two time points.

Bivariate analyses were also conducted among patients with a documented DNACPR and/or DNI order to determine patient and clinical characteristics associated with having a DNACPR and/or DNI order. Outcomes were assessed for all patients with a DNACPR and/or DNI order as well as between the early and late cohort. All analytic assumptions were verified, and analyses were performed using SAS v9.1 (SAS Institute, Cary, North Carolina).

## Results

Among 1375 hospitalized patients with COVID-19, 19% (*n* = 258) of all patients had a documented DNACPR and/or DNI order (Table 1). The mean age of all Covid-19 patients was 56 years old, and the mean age of COVID-19 patients with a DNACPR and/or DNI order was 74 years old. The study population was 50% male and 50% female, 42% white and 43% black. A total of *n* = 158 (12%) patients in the cohort died during hospitalization.Table 2.

**Table 1:** Demographics and Characteristics of Documented DNR Orders in COVID-19 Patients.

	All Patients	Patients with DNR/DNI Orders	Patients with no code status limitations	Bivariate	Multivariate Analysis	Multivariate Analysis
				p-value	p-value	Odds Ration (95% CI)
All COVID-19 patients	N = 1375	N = 258 (18.8%)	N = 1117 (81.2%)			
Age (mean, standard deviation)	55.6 (18.0)	73.8 (13.1)	51.4 (16.3)	**<0.0001**	1.12 (1.09, 1.14)	**<0.0001**
Gender						
Female	679 (49.4)	117 (17.2)	562 (82.8)			
Male	696 (50.6)	141 (20.3)	555 (79.7)	0.1506		
Race						
White	574 (41.8)	127 (22.1)	447 (77.9)	**0.0002**	Reference	
Black	591 (43.0)	112 (19.0)	479 (81.0)		0.64 (0.41, 0.98)	0.0655
Other	210 (15.3)	19 (9.0)	191 (91.0)		1.18 (0.50, 2.82)	0.3710
Ethnicity						
Hispanic	383 (27.9)	36 (9.4)	347 (90.6)	**<0.0001**	1.42 (0.67, 3.00)	
Non-Hispanic	992 (72.2)	222 (22.4)	770 (77.6)		Reference	0.3585
Acute stroke	51 (3.7)	19 (37.3)	32 (62.8)	**0.0006**	1.14 (0.55, 2.33)	0.7278
P.E.	19 (1.4)	2 (10.5)	17 (89.5)	0.3544		
Mean days in the hospital	6 (3, 13)	11 (5, 17)	6 (3, 11)	**<0.0001**		
White Cell Count (k/cumm)	7.8 (5.7, 10.5)	9.2 (6.7, 12.9)	7.6 (5.5, 10.1)	**<0.0001**	1.11 (1.06, 1.16)	**<0.0001**
Lymphocyte Count (k/cumm)	1.0 (0.7, 1.5)	1.0 (0.7, 1.4)	1.1 (0.7, 1.6)	0.0929		
Hemoglobin	0.2 (0.1, 0.7)	0.2 (0.1, 0.9)	0.2 (0.1, 0.7)	0.2104		
Procalcitonin	0.2 (0.1, 0.7)	0.2 (0.1, 0.9)	0.2 (0.1, 0.7)	0.2104		
LDH	341 (272, 432)	338 (267, 414)	343 (273, 436)	0.3856		
AST	38 (26, 59)	37 (26, 54)	39 (27, 60)	0.5037		
ALT (units/L)	35 (21, 60)	28 (17, 45)	38 (21, 63)	**<0.0001**	1.00 (1.00,1.00)	0.5426
Pao2: FiO2 ratio (fpr intubated patients)	0.7 (0.5, 44.5)	0.7 (0.5, 45)	0.6 (0.5, 42)	0.5364		
D-Dimer	1.2 (0.7, 2.2)	1.3 (0.7, 2.5)	1.1 (0.6, 2.2)	0.2309		
Creatinine	0.9 (0.7, 1.3)	1.0 (0.8, 1.7)	0.8 (0.7, 1.1)	**<0.0001**	0.92 (0.81, 1.05)	0.2082
Covid Cohort						
Early	1006 (73.2)	211 (21.0)	795 (79.0)	**0.0005**	2.08 (1.28, 3.41)	**0.0034**
Later	369 (26.8)	47 (12.7)	322 (87.3)		Reference	

Bivariate analysis values are means (standard deviations) for age, medians (IQRs) for other continuous variables, and frequencies (percentages) for categorical variables; p-values are from t-tests, Wilcoxon rank-sum tests, and Chi-Square tests (verified with Fisher’s Exact test when expected cell counts are <5 for <20% of cells), respectively. Multivariate analysis values are odds ratios (95% confidence intervals) for being in the DNR/DNI group, with p-values from multivariate logistic regression model.

**Table 2:** Patient and Clinical Characteristics at the Time of Documented DNR Order Limiting Life Prolonging Interventions in COVID-19 Patients during Early and Late COVID-19 Hospital Admissions.

	All Patients	Early Covid-19 (March – June 2020)	Late Covid-19 (July – October 2020)	P-value
**ALL PATIENTS**	**N = 1375**	**N = 1006 (73.2%)**	**N = 369 (26.8%)**	
Age	55.6 (18.0)	56.7 (17.5)	52.7 (19.0)	**0.0002**
Gender				
Female	679 (49.4)	494 (72.8)	185 (27.3)	0.7350
Male	696 (50.6)	512 (73.6)	184 (26.4)	
Race				
White	574 (41.8)	397 (69.2)	177 (30.8)	**0.0002**
Black	591 (43.0)	466 (78.9)	125 (21.1)	
Other	210 (15.3)	143 (68.1)	67 (31.9)	
Ethnicity				
Hispanic	383 (27.9)	276 (72.1)	107 (27.9)	0.5670
Non-Hispanic	992 (72.2)	730 (73.6)	262 (26.4)	
ICU Utilization	591 (43.0)	428 (72.4)	163 (27.6)	0.5888
Pneumonia	132 (9.6)	103 (78.0)	29 (22.0)	0.1845
Sepsis	80 (5.8)	62 (77.5)	18 (22.5)	0.3671
Acute Stroke	51 (3.7)	35 (68.6)	16 (31.4)	0.4563
In Hospital Mortality	158 (11.5)	129 (81.7)	29 (18.4)	**0.0105**
**DNR/DNI PATIENTS**	**N = 258**	**N = 211 (81.8%)**	**N = 47 (18.2%)**	
Age (mean)	73.8 (13.1)	73.7 (13.0)	74.6 (13.8)	0.6439
Gender				
Female	117 (45.4)	99 (84.6)	18 (15.4)	0.2830
Male	141 (54.7)	112 (79.4)	29 (20.6)	
Race				
White	127 (49.2)	100 (78.7)	27 (21.3)	0.1850
Black	112 (43.4)	97 (86.6)	15 (13.4)	
Other	19 (7.4)	14 (73.7)	5 (26.3)	
Ethnicity				
Hispanic	36 (14.0)	30 (83.3)	6 (16.7)	0.7950
Non-Hispanic	222 (86.0)	181 (81.5)	41 (18.5)	
Palliative Care Consult	171 (66.3)	151 (88.3)	20 (11.7)	**0.0001**
ICU Utilization	167 (64.7)	138 (82.6)	29 (17.4)	0.6311
Pneumonia	34 (13.2)	29 (85.3)	5 (14.7)	0.5692
Sepsis	35 (13.6)	27 (77.1)	8 (22.9)	0.4443
ECMO	7 (2.7)	2 (28.6)	5 (71.4)	**0.0002**
Acute stroke	19 (7.4)	14 (73.7)	5 (26.3)	0.3420
Prone position	51 (19.8)	44 (86.3)	7 (13.7)	0.3535
Paralytics	66 (25.6)	57 (86.4)	9 (13.6)	0.2637
Hospital day of DNR/DNI Order	4 (1, 12)	4 (1, 11)	4 (1, 15)	0.5375
Multiple code status changes	67 (26.7)	63 (91.3)	6 (8.7)	**0.0167**
Level of pulmonary ventilator support				
N/A	43 (16.9)	36 (83.7)	7 (16.3)	
Room Air	43 (16.9)	35 (81.4)	8 (18.6)	
Nasal Cannula	61 (23.9)	52 (85.3)	9 (14.8)	
High Flow NC	34 (13.3)	27 (79.4)	7 (20.6)	
Bipap/Cpap	6 (2.4)	3 (50.0)	3 (50.0)	
Mech Vent	68 (26.7)	57 (83.8)	11 (16.2)	0.4087
Dialysis				
New	23 (52.3)	21 (91.3)	2 (8.7)	
Chronic	21 (47.7)	20 (95.2)	1 (4.8)	0.6051
In Hospital Mortality	147 (57.0)	121 (82.3)	26 (17.7)	0.7996
***Labs***				
Pao2: FiO2 ratio (for intubated patients)	0.7 (0.5, 45.0)	0.7 (0.5, 45.0)	0.8 (0.5, 30.0)	0.6781
White cell count	9.2 (6.7, 12.9)	8.9 (6.6, 12.8)	9.9 (6.8, 12.9)	0.4104
Lymphocyte count	1.0 (0.7, 1.4)	1.0 (0.7, 1.5)	0.9 (0.6, 1.2)	0.2297
Platelet count	212 (151, 289)	217 (160, 291)	197 (142, 260)	0.1545
Hemoglobin	0.2 (0.1, 0.9)	0.3 (0.1, 1.1)	0.2 (0.1, 0.4)	0.2007
C-Reactive Protein	8.1 (4.7, 13.6)	8.6 (4.7, 14.1)	7.3 (5.0, 11.9)	0.4527
Procalcitonin	0.2 (0.1, 0.9)	0.3 (0.1, 1.1)	0.2 (0.1, 0.4)	0.2007
LDH	338 (267, 414)	336 (272, 414)	343 (252, 414)	0.7196
AST	37 (26, 54)	37 (26, 55)	37 (27, 52)	0.9617
ALT	28 (17, 45)	27 (17, 46)	31 (17, 43)	0.6531
Total Bili	0.6 (0.4, 0.7)	0.6 (0.4, 0.7)	0.5 (0.4, 0.7)	0.4475
Creatinine	1.0 (0.8, 1.7)	1.0 (0.8, 1.6)	1.1 (0.8, 1.8)	0.3458
D-Dimer	1.3 (0.7, 2.5)	1.3 (0.7, 2.3)	1.5 (0.8, 3.0)	0.1950
Ferritin	570 (299, 1057)	579 (293, 1082)	529 (330, 988)	0.9422
Had Cardiopulmonary resuscitation prior to DNR/DNI Order	2 (20.0)	2 (100)	0 (0)	0.5982
If coded - Survived Code (successful)	8 (80.0)	7 (87.5)	1 (12.5)	0.5982
Was code status ever change back to full code	2 (3.0)	2 (100)	0 (0)	0.6578
**COVID-19 Treatments**				
Remdesivir	18 (12.0)	8 (44.4)	10 (55.6)	**0.0004**
Dexamethasone	43 (16.9)	24 (55.8)	19 (44.2)	**<0.0001**
Other Steroids	48 (18.8)	43 (89.6)	5 (10.4)	0.3448
Tocilizumab	25 (9.9)	21 (84.0)	4 (16.0)	0.9175
Convalescent Plasma	16 (6.3)	12 (75.0)	4 (25.0)	0.6916

Values are means (standard deviations) for age, medians (IQRs) for other continuous variables, and frequencies (percentages) for categorical variables; p-values are from t-tests, Wilcoxon rank-sum tests, and Chi-Square tests (verified with Fisher’s Exact test when expected cell counts are <5 for <20% of cells), respectively.

### Predictors of having a DNACPR and/or DNI Order

In bivariate analysis, age and racial differences were found between patients with a DNACPR order. Patients who were hospitalized during the early time period of the pandemic were more likely to have a DNACPR order compared to patients hospitalized in the later time period of the pandemic (*p* =< 0.01). In multivariable analysis, age (older) *p* =< 0.01, OR 1.12 and hospitalization during the early time period of the pandemic *p* = 0.01, OR 2.08, were associated with having a DNACPR order (Table 1).

### Clinical interventions in patients with a DNACPR and/or DNI order

Among the *n* = 1375 patients hospitalized with COVID-19, 43% of patients (*n* = 591) spent time in the ICU. Palliative care consultation occurred in 66% of all patients hospitalized with COVID-19 (*n* = 167). Among the *n* = 258 patients with a DNACPR and/or DNI order, palliative care consultation occurred more often in the early COVID-19 cohort (88%, *n* = 171 versus 12%, *n* = 20, *p* =< 0.01) (Table 3). Medical interventions, including ICU (*p* = 0.63) and level of ventilator support (*p* = 0.41) did not differ between cohorts. COVID-19 treatments including Remdesivir (*p* = 0.01) and Dexamethasone (*p* =< 0.01) increased during the late cohort.

**Table 3:** Outcomes Associated with DNR/ DNI Orders in COVID-19 Patients.

	All patients with DNR/DNI Order N = 258	Early Cohort N = 211 (81.8%)	Late Cohort N = 47 (18.2%)	P-value
In Hospital mortality	147 (57.0)	121 (82.3)	26 (17.7)	0.7996
Race				
White	127 (49.2)	100 (78.7)	27 (21.3)	0.1850
Black	112 (43.4)	97 (86.6)	15 (13.4)	
Other	19 (7.4)	14 (73.7)	5 (26.3)	
Median hospital day of mortality	13 (8, 21)	14 (8, 21)	11 (5, 17)	0.1889
Median day from DNR/DNI Order to death	5 (2, 9)	5 (2, 11)	2 (1, 5)	**0.0028**
DNR/DNI order at time of CMO	106 (41.1)	91 (85.9)	15 (14.1)	0.1576
Mean day of hospitalization of transition to comfort measures only	10 (5, 16)	9 (4, 16)	10 (7, 15)	0.8550
Had cardiopulmonary resuscitation prior to code status order	2 (20.0)	2 (100)	0 (0)	0.5982
Palliative care consult prior to first code status order	65 (40.1)	58 (89.2)	7 (10.8)	0.7561
Survived to discharge with code status order	102 (39.5)	80 (78.4)	22 (21.6)	0.2594
Discharge disposition of patients with code status order who survived				
Expired/Hospice	9 (8.8)	8 (88.9)	1 (11.1)	0.3471
Home/routine/AMA	34 (33.3)	24 (70.6)	10 (29.4)	
Other/law enforcement	59 (57.8)	48 (81.4)	11 (18.6)	

Values are frequencies (percentages) for categorical variables and medians (IQRs) for continuous variables, with p-values from Chi-Square tests (verified with Fisher’s Exact test when expected cell counts are <5 for <20% of cells) and Wilcoxon rank-sum tests, respectively.

### Outcomes in COVID-19 patients with a DNACPR and/or DNI order

**Fig. 1:**
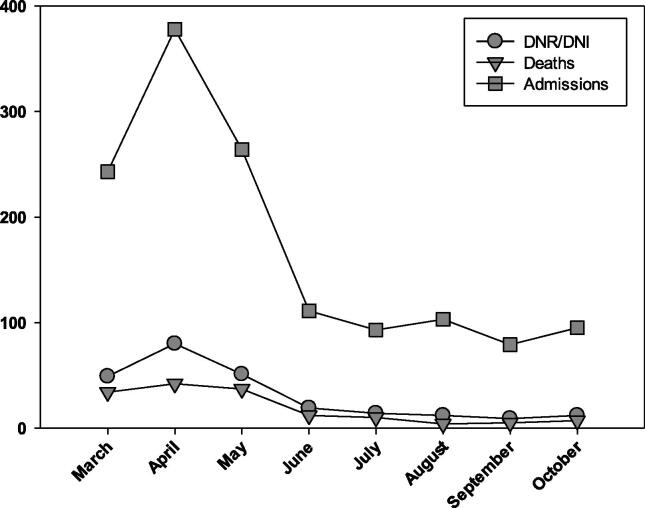
DNR Orders among patients hospitalized with COVID-19.

Between the early and late hospital admission cohorts, bivariate analysis found few differences between outcomes including mean day of hospital mortality (*p* = 0.18), or discharge disposition (*p* = 0.35) (Table 3). The only outcomes which differed between the early and late cohort was median day from DNACPR and/or DNI order to death and in hospital mortality (Fig. 1). The early cohort median was 5 days between DNACPR and/or DNI order and death and the late cohort median was 2 days between DNACPR and/or DNI order and death (*p* =< 0.01). Among patients with a DNACPR and/or DNI order, in hospital mortality did not differ between cohorts, *p* = 0.8.

## Discussion

This study found a higher prevalence of DNACPR and/or DNI orders for patients hospitalized during the beginning of the COVID-19 pandemic versus a later time period during the first wave of the pandemic. Additionally, this study found that DNACPR and/or DNI orders were written earlier in the patient’s hospitalization in the beginning of the COVID-19 pandemic versus the later time period. The similarity between cohort characteristics, including similar lab values and clinical interventions suggests that differences in DNACPR and/or DNI order utilization may be due to a change in clinical practice between the two time periods and not due to a difference in how sick the patients were in each cohort.

Patient and physician assumptions and knowledge about COVID-19 prognosis and resuscitation outcomes evolved during the first year of the pandemic. Additionally, COVID-19 treatments including Remdesivir became available and provided a glimmer of hope for improved patient recovery; however, the use of Remdesivir has been found to be most effective in patients who do not receive oxygen therapy, thus the use of Remdesivir does not clinically explain the lower prevalence of DNACPR/ DNI orders in critically ill patients as these patients were not likely to be responders to Remdesivir.^27^ Given the lack of a clinical explanation for differences in prevalence and timing of DNACPR/ DNI orders between the cohorts, we are left to examine the complex array of factors that are associated with the timing and conclusion of code status discussions, including physician communication behaviors, patient demographics, and staffing model of the intensive care unit.^28^, ^29^, ^30^, ^31^, ^32^ All of these variables were affected in various ways by the pandemic. The role of these considerations in code status decisions may have been a significant driver of changing code status ordering practices between the two cohorts.

The COVID-19 pandemic introduced new variables which potentially impacted code status decisions, some of which may have played a greater role earlier in the pandemic. The early pandemic was characterized by fear of disease transmission. Healthcare workers are not accustomed to considering personal health risks of providing medical care, but shortages of personal protective equipment, such as masks and gowns, brought this concern to the forefront. The initial lack of evidence and understanding of the disease course, prognostic uncertainty, and resource shortages of common items such as masks and gowns created challenges in adhering to usual standards of care in some hospitals, although it is unclear whether this occurred at the study hospitals or whether this impacted code status decisions.^33^, ^34^, ^35^, ^36^

Furthermore, the possibility of shortages of medical equipment, such as ventilators, may have sparked concerns among providers about effective utilization of such equipment and may have influenced the approach or timing of conversations with patients (or their surrogates) when patients were perceived as poor candidates for such interventions. Ventilator allocation policies were not invoked in the study health systems but an impact of uncertainty about ventilator supplies on the practice of code status deliberations cannot be ruled out. As part of previously developed crisis standards of care, some considered the possibility of using DNACPR and/or DNI orders as a criterion for exclusion from ventilator eligibility, but many systems saw this as problematic, and did not ultimately include it as a criterion. It is also unclear how an increased workload for clinicians may have impacted code status deliberations early in the pandemic.

Shortages of medical equipment, fear of disease transmission, and increased workload for clinicians likely resulted in physician’s approaching treatment decisions for COVID-19 patients through the lens of triage rules traditionally used in disaster and war medicine. The crux of the shift from the standard practice of medicine to implementing triage or disaster protocols results from shifting from “doing what is best for the individual patient to doing the greatest good for the largest number of people”.^37^ Part of triage is to “sort patients” into categories based on disease severity from top priority to no priority. Under the traditional disaster triage categories, patients who have such extensive injuries that they are likely to die are not treated with priority. It is possible that patients were being provided the opportunity to change their code status to DNACPR and/or DNI earlier and more frequently during the early part of the pandemic as a part of triaging patients.

Another consideration is that communication with patients and families was drastically altered by isolation procedures and the halting of all or nearly all family visitation.^38^ The lack of family visitation meant that family meetings with clinicians to discuss prognosis and treatment could not be held in person. Such in person contact may help families come to terms with the patient’s critical illness and in some cases may facilitate understanding about the appropriateness of a change in code status.

Although there were differences in overall mortality between the two study cohorts, there was not a difference in mortality between cohorts for patients with DNACPR and/or DNI orders. This is an important outcome given past studies which have suggested worse outcomes in COVID-19 patients with DNACPR and/or DNI orders.^39^, ^40^, ^41^ The higher prevalence of palliative care consultation in the early time period may have been a covariate born of the same factors which led to more and earlier DNACPR and/or DNI orders. However, it is unclear whether the higher prevalence of palliative care consultation in the earlier surge directly contributed to the higher prevalence of DNACPR and/or DNI orders.

Lastly, this study found racial differences in code status orders with white patients having a significantly higher prevalence of DNACPR and/or DNI code status orders than black patients, although this finding lost significance in multivariable analysis. This finding is consistent with prior studies which have found racial differences in end-of-life care and decision making including black patients receiving more intensive treatments near the end of life and having a higher prevalence of DNACPR and/or DNI orders than white patients.^42^, ^43^ Some studies suggest that religious beliefs about end-of-life care may play a role in treatment preferences, while other studies suggest that this is a result of past unjust treatment of minority groups by the health care system.^44^ We did not find evidence that early versus late admission affected the relationship between race and DNACPR and/or DNI orders, suggesting racial differences remained consistent across pandemic time periods.

This study includes several limitations. First, this was a retrospective cohort thus, making determinations about the association between documented code status and reasons behind medical decision making is challenging. However, code status orders provide an opportunity to study changes in medical decision-making regarding life sustaining therapies because these orders are well-documented in the medical record. Second, this study was conducted at two institutions in the Midwest, United States, and may not be representative of other institutional practices during the early pandemic. This study did not evaluate whether changes in code status ordering patterns applied only to patients positive for COVID-19 or whether this phenomenon was present in other disease courses during the time of the study. Lastly, the inclusion criteria for this study included all patients with a positive PCR COVID-19 test and a discharge diagnosis of COVID-19, therefore it is possible that patients may have been severely ill due to other reasons outside of COVID-19 which may have prompted a code status discussion. Future studies are needed to determine the possibility of a temporal variation outside of the COVID-19 pandemic and whether the phenomenon discovered in this study occurred at other institutions.

## Conclusions

There was a higher prevalence of DNACPR and/or DNI orders earlier in the hospital course for patients hospitalized early in the pandemic versus once the first wave of cases had ebbed, despite similarities in clinical characteristics, medical interventions, and in hospital mortality. Changes in clinical care between cohorts may be due to fear of resource shortages and changes in knowledge about COVID-19. To inform ethical practice during public health crises, these changes should be examined further to better understand the factors which impact code status orders and other decisions regarding life prolonging therapies during the COVID-19 pandemic.

## Funding

None.

## Declaration of Competing Interest

The authors declare that they have no known competing financial interests or personal relationships that could have appeared to influence the work reported in this paper.
